# Pustular dermatosis of the scalp due to topical minoxidil 5%

**DOI:** 10.11604/pamj.2018.30.83.15384

**Published:** 2018-05-29

**Authors:** Ouiam El Anzi, Badreddine Hassam

**Affiliations:** 1 Service de Dermatologie et Vénérologie, Centre Hospitalier Universitaire Ibn Sina, Faculté de Médecine et de Pharmacie, Université Mohammed V, Rabat, Maroc

**Keywords:** Minoxidil, allergic contact dermatitis, pustules

## Image in medicine

Topical minoxidil, the only approved treatment for female pattern hair loss (FPHL), has been associated with scalp allergic contact dermatitis (ACD). We report the case of a female patient who developed ACD from minoxidil solution. A 30-year-old women presented with pruriginous and pustular lesions over an erythematous area on the scalp and forehead. These lesions had appeared after 1 week of treatment with 5% minoxidil solution (Foligan®)[minoxidil 5%, ethanol, propylene glycol and purified water], for androgenetic alopecia. Physical examination revealed multiple millimetric papulovesicles and papulopustules over an erythematous oedematous area on the frontal scalp and forehead. The patient refused to have a biopsy taken. With these clinical lesions a diagnosis of pustular contact dermatitis was made. Minoxidil solution was stopped and topical corticosteroid therapy twice a day was started. A complete clearance of the lesions could be observed after 5 days. Topical 2,6-diamino-4-piperidinopyrimidine 1-oxide (minoxidil) has been shown to be effective in the treatment of androgenetic alopecia for over 15 years. Minoxidil 5% solution has a favorable safety profile, with adverse effects limited to the site of application (5.7% of patients) and signed by pruritus, erythema, scaling and dryness. The most common causes of these symptoms include irritant contact dermatitis, allergic contact dermatitis, or exacerbation of seborrheic dermatitis. Although cases of pustular allergic contact dermatitis have been anecdotally reported. We conclude that our case is interesting in that it represents an uncommon reaction to a drug used very frequently in dermatology.

**Figure 1:**
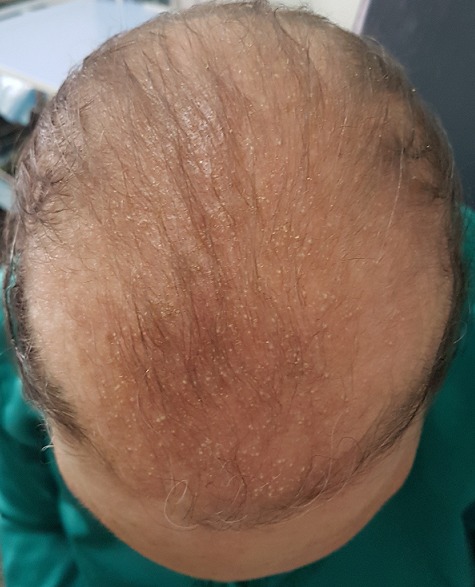
Pustules and vesicles over an erythematous area on the frontal scalp

